# Impact of cultivation duration and methods on soil physicochemical properties, ginseng growth, and arbuscular mycorrhizal fungi community structure

**DOI:** 10.3389/fmicb.2025.1628889

**Published:** 2025-08-19

**Authors:** Jinlong Wang, Zhihui Kan, Xiaole Zhang, Boya Yang, Yuxuan Dong, Bo Wang, Chunjuan Wang

**Affiliations:** ^1^College of Science, Beihua University, Jilin, China; ^2^Traditional Chinese Medicine Biotechnology Innovation Center in Jilin Province, Beihua University, Jilin, China

**Keywords:** *Panax ginseng*, arbuscular mycorrhizal fungi, soil properties, ginsenoside accumulation, continuous cropping, microbial diversity

## Abstract

**Introduction:**

Ginseng (*Panax ginseng* C. A. Meyer) is a widely cultivated medicinal plant valued for its bioactive ginsenosides, which are influenced by soil conditions and microbial interactions. However, the long-term effects of different cultivation durations and methods on soil physicochemical properties, arbuscular mycorrhizal fungi (AMF) communities, and ginsenoside accumulation remain largely unexplored.

**Methods:**

This study investigates the relationships between soil characteristics, AMF community structure, and ginseng quality under different cultivation treatments using high-throughput sequencing, soil property analysis, and canonical correspondence analysis (CCA).

**Results:**

The results indicate that long-term ginseng cultivation significantly reduces soil moisture, organic matter, and nitrate nitrogen contents. Soil pH initially increased and subsequently declined over the cultivation period, and the contents of phosphorus and potassium elements show a fluctuating downward trend with the increase in cultivation years. AMF community composition varied across cultivation years, with *Glomus* and *Paraglomus* dominating in later stages, potentially influencing plant nutrient uptake and secondary metabolite synthesis. CCA shows that nitrate nitrogen, total nitrogen, and total phosphorus are positively correlated with AMF community structure, while soil moisture content is negatively correlated. Additionally, ginsenoside accumulation was significantly affected by cultivation conditions, with AMF interactions likely modulating secondary metabolism.

**Discussion:**

These findings provide valuable insights into microbial-mediated soil health management and offer strategies for optimizing ginseng cultivation practices to enhance plant performance and bioactive compound production.

## 1 Introduction

Ginseng (*Panax ginseng* C. A. Meyer) is a highly valued medicinal herb widely cultivated for its pharmacologically active compounds, particularly ginsenosides, which exhibit immunomodulatory, anti-inflammatory, and antioxidative properties (Guo et al., 2021; Valdés-González et al., 2023). The growth, development, and secondary metabolite accumulation of ginseng are profoundly influenced by soil conditions and microbial communities, especially arbuscular mycorrhizal fungi (AMF). However, continuous cropping and different cultivation methods significantly alter soil physicochemical properties, which in turn affect microbial diversity, plant growth performance, and metabolite biosynthesis (Gupta et al., 2022; Li et al., 2021). Despite increasing research efforts, the intricate interactions among soil characteristics, AMF diversity, and ginseng quality remain largely unexplored, necessitating further investigations into these complex relationships.

Previous studies have demonstrated that soil degradation, particularly the depletion of organic matter, nitrogen, and phosphorus availability, poses a major challenge in long-term ginseng cultivation (Liu et al., 2022; Lv et al., 2025; Miao et al., 2025). Intensive farming practices and prolonged monoculture can lead to shifts in soil pH, electrical conductivity (EC), and nutrient bioavailability, consequently influencing microbial community structure and functional diversity (Li et al., 2018). AMF, as key microbial symbionts, play a crucial role in soil fertility maintenance and plant resilience to biotic and abiotic stresses. However, the extent to which different cultivation durations and methods impact AMF community assembly and its ecological implications for ginseng production remain poorly understood.

In addition to soil microbial dynamics, the accumulation of secondary metabolites in ginseng is closely related to environmental conditions. Ginsenosides, the major bioactive constituents of ginseng, have been shown to be influenced by soil nutrient availability and microbial interactions (Fang et al., 2025; Li et al., 2020; Yang et al., 2022). AMF, in particular, have been found to affect secondary metabolism in medicinal plants by modulating root exudation patterns, altering nutrient acquisition strategies, and stimulating plant defense pathways (Thokchom et al., 2023). The mechanisms by which AMF influence ginsenoside biosynthesis may involve modifications in host plant signaling pathways, changes in root architecture, and alterations in soil microbial community composition (Mu et al., 2024; Ng et al., 2024). However, the specific molecular and biochemical processes underlying AMF-induced ginsenoside production remain largely unknown, requiring further research into the functional roles of mycorrhizal symbiosis in medicinal plant production.

Furthermore, the effects of different cultivation practices on soil health and microbial communities require a more comprehensive understanding. Continuous monoculture has been linked to increased pathogen accumulation, nutrient depletion, and microbial dysbiosis, which may negatively impact ginseng growth and quality (Rao et al., 2021; Zhang et al., 2020). In contrast, intercropping, organic fertilization, and natural forest cultivation are considered more sustainable, as they can mitigate soil degradation and preserve microbial diversity. AMF as key symbiotic partners of most terrestrial plants, play a pivotal role in enhancing nutrient uptake, promoting stress tolerance, and modulating plant secondary metabolism (Fan et al., 2024; Wahab et al., 2023). Although previous studies have highlighted the beneficial roles of AMF in ginseng and other medicinal plants (Thokchom et al., 2023), the ecological and functional dynamics of AMF communities under varying cultivation durations and systems remain poorly characterized.

Importantly, the accumulation of pharmacologically active ginsenosides in ginseng is strongly influenced by soil nutrient status and microbial associations, particularly AMF. Mechanistic studies suggest that AMF may regulate secondary metabolism by enhancing phosphorus acquisition, altering root exudation patterns, and inducing defense-related signaling pathways (Mu et al., 2024; Ng et al., 2024). However, existing evidence is mostly indirect, and the relationships between AMF diversity, soil properties, and ginsenoside biosynthesis require further investigation in field-grown systems. To address these knowledge gaps, this study systematically investigates the effects of different cultivation durations (1–4 years) and cultivation systems (forest vs. garden) on soil physicochemical properties, AMF community composition, and ginsenoside accumulation in ginseng. By integrating high-throughput sequencing, soil property analysis, and canonical correspondence analysis (CCA), we aim to uncover the key environmental and microbial factors driving ginseng quality under different management regimes. Specifically, we test the following hypotheses:

*H1*: ginseng cultivation duration and method significantly alter the structure and diversity of AMF communities.

*H2*: soil physicochemical properties, particularly nitrogen and phosphorus availability, are key drivers shaping AMF community composition.

*H3*: changes in AMF community structure are associated with variations in ginsenoside accumulation in ginseng roots.

Our findings are expected to enhance the ecological understanding of AMF-plant–soil interactions in ginseng cultivation and provide guidance for optimizing sustainable production strategies based on microbial community management.

## 2 Materials and methods

### 2.1 Study site and plant material

This study was conducted in Songyuan City, Jilin Province, China (44°24′N, 124°49′E), a major ginseng-producing region characterized by a temperate monsoon climate with cold, dry winters and warm, humid summers. The mean annual temperature ranges from 4.6°C to 6.4°C, and average annual precipitation is approximately 400 mm, with most rainfall concentrated between June and August. The regional soil type is classified as black soil (Mollisol), known for its high fertility and organic matter content, but susceptible to degradation under continuous cropping.

To evaluate the influence of different cultivation systems and durations on ginseng-associated soil environments and microbial communities, we selected five representative cultivation treatments: R1–R4 (garden-cultivated ginseng): ginseng plants cultivated in managed plantation fields for 1 to 4 consecutive years, respectively. These represent typical short-term continuous cropping systems with increasing cultivation intensity. R5 (forest-cultivated ginseng): ginseng cultivated for 20 years in a managed plantation field. The 20-year-old forest-cultivated ginseng (R5) was included to compare ecological outcomes between short-term, high-intensity garden cultivation and long-term, low-input forest growth, while assessing cumulative effects of perennial understory growth on soil, AMF communities, and ginsenoside accumulation—reflecting real-world distinctions between the two practices.

### 2.2 Experimental design and soil sampling

A total of 25 root-adhering soil samples were collected from five treatment groups: R1 (1 year), R2 (2 years), R3 (3 years), R4 (4 years), and R5 (naturally grown ginseng). Each sample was obtained from the root-adhering soil of three randomly selected plants within a plot and thoroughly mixed to form a composite sample. Five replicates were collected per treatment group.

The soil samples were kept in a refrigerated box during transport to the laboratory, where they were carefully homogenized and sieved through a 2-mm mesh (Gou et al., 2015). Each sample was split into three aliquots. The first aliquot was stored at −20°C for DNA extraction. The second aliquot was employed to assess soil moisture: after drying at 105°C for 48 h, soil moisture content (MC) was determined by weighing. The third aliquot, following 15 days of air-drying, was utilized to analyze soil pH, electrical conductivity, total nitrogen (TN), ammonium nitrogen (AN), nitrate nitrogen (NN), available phosphorus (AP), total phosphorus (TP), available potassium (AK), and total potassium (TK). MC was determined using the gravimetric method. pH was measured in a 1:2.5 (w/v) soil-to-water suspension with a pH meter. EC was recorded using a conductivity meter. Soil organic matter (OM) was analyzed via dichromate oxidation, TN, AN, NN, AP, TP, AK, and TK were determined using standard soil analysis protocols (Maestre et al., 2022).

### 2.3 DNA extraction and high-throughput sequencing

Total soil DNA was extracted from 0.5 g of rhizosphere soil using the PowerSoil DNA Isolation Kit (MoBio Laboratories, United States) following the manufacturer’s protocol. The SSU rRNA gene region specific to AMF was amplified using AMF-specific primers NS31 and AML2. The amplicons were purified, pooled in equimolar concentrations, and sequenced on the Illumina MiSeq platform (Illumina, San Diego, CA, USA) by Majorbio BioPharm Technology Co., Ltd. (Shanghai, China).

Raw sequencing data were processed using QIIME2, including quality filtering, chimera removal, and OTU clustering at a 97% similarity threshold. Taxonomic classification was performed against the MaarjAM AMF reference database (Wang et al., 2021). Alpha diversity indices (Shannon, Simpson, Chao1, and ACE) and beta diversity (PCoA) were calculated to evaluate community richness and structure.

### 2.4 Plant sampling and Ginsenoside analysis

To assess the influence of cultivation duration and method on ginseng growth and ginsenoside accumulation, plant samples were collected concurrently with rhizosphere soil from each treatment group (R1–R4 and R5). In each plot, three healthy and representative ginseng plants were randomly selected and excavated with minimal root disturbance (Wang et al., 2024). These plants were then combined to form one composite sample, and five biological replicates were collected per group (*n* = 5).

Immediately after harvest, plants were gently rinsed with distilled water to remove soil particles and surface debris. The samples were then blotted dry, sealed in clean polyethylene bags, and stored on ice during transport to the laboratory. For ginsenoside analysis, fresh root samples were first stored at −80°C, then freeze-dried (lyophilized) and ground into a fine powder using a sterilized mechanical grinder (Zhang et al., 2012).

Total ginsenoside content was determined using a spectrophotometric method following ethanol extraction (Liu et al., 2018). Specifically, 0.5 g of powdered root material was extracted with 10 mL of 70% ethanol in an ultrasonic water bath at 40°C for 60 min. The extract was centrifuged at 8000 rpm for 10 min, and the supernatant was collected. The total ginsenoside content in the extract was quantified by colorimetric reaction with vanillin-perchloric acid reagent and measured at 560 nm using a UV–visible spectrophotometer (Sun et al., 2009).

### 2.5 Statistical analysis

One-way analysis of variance (ANOVA) with Tukey’s *post hoc* test was used to assess differences in soil physicochemical properties and AMF diversity among treatment groups. Spearman’s correlation analysis was performed to determine relationships between AMF diversity indices, soil properties, and ginseng growth parameters. Canonical correspondence analysis (CCA) was conducted to explore the influence of soil variables on AMF community composition. PCoA was performed based on the Bray–Curtis dissimilarity matrix of AMF community composition data. The analysis was conducted using R software (version 4.1.0), and the resulting ordination plots were used to visualize the relationships and differences among AMF communities under different treatments. Statistical analyses were performed using R software (version 4.1.0).

## 3 Results

### 3.1 Variations in soil physicochemical properties under different cultivation years and methods

Soil physicochemical properties exhibited significant changes with different cultivation years and methods (Table 1). MC decreased from 34.55% in R1 to a minimum of 20.15% in R3, followed by a slight recovery in R4 (24.05%). The forest understory (R5) had a slightly higher MC than R3 but showed no significant difference from R3 and R4. Soil pH peaked at 6.59 in R3 and was lowest in R5 (5.95), indicating that cultivation methods had a more pronounced effect on pH than cultivation years. Electrical conductivity (EC) varied significantly, with R4 (291.2 μs/cm) exhibiting mild salinization, while R5 (207.8 μs/cm) was comparable to R2 and R3. Organic matter (OM) fluctuated, peaking at 102.03 g/kg in R2 and dropping to a minimum of 23.96 g/kg in R3. R5 had slightly higher OM than R2 but was significantly different from R4 (Table 1).

**Table 1:** Effect of different cultivation durations and methods on soil physicochemical properties in ginseng cultivation.

Treatment	pH	EC (μs/cm)	OM (g/kg)	AN (mg/kg)	NN (mg/kg)	AK (mg/kg)	AP (mg/kg)	TN (g/kg)	TK (g/kg)	TP (g/kg)
R1	6.51 ± 0.16ab	282.8 ± 49.4a	67.85 ± 16.2a	35.19 ± 7.54a	94.73 ± 55.4a	114.68 ± 30.3a	2.334 ± 1.03a	1.434 ± 0.53a	114.5 ± 24.1a	1.56 ± 1.06a
R2	6.47 ± 0.13ab	208.2 ± 28.4b	102 ± 17.8ab	32.42 ± 3.69a	39.31 ± 4.39b	148.98 ± 27.5ab	4.196 ± 1.46ab	0.816 ± 0.16b	151.2 ± 31.5ab	0.582 ± 0.38a
R3	6.59 ± 0.09a	205.6 ± 21.9b	23.96 ± 11.2c	20.62 ± 3.04c	33.11 ± 12.7b	91.66 ± 13.4ab	0.556 ± 0.36b	0.57 ± 0.28bb	84.66 ± 11.1ab	1.064 ± 0.34a
R4	6.43 ± 0.10b	295.2 ± 16.9c	47.39 ± 6.7c	33.75 ± 2.37a	50.49 ± 17.3b	133.06 ± 22.6b	4.528 ± 5.37bc	0.684 ± 0.09b	152.04 ± 31.3b	1.286 ± 0.17b
R5	5.95 ± 0.33c	207.8 ± 9.9bd	104.2 ± 21.2b	42.61 ± 5.91b	51.11 ± 14.4ab	101.56 ± 36.1c	3.528 ± 0.18c	0.888 ± 0.1c	90.64 ± 42.9ab	1.676 ± 0.45b

Values are expressed as mean ± SD. Different lowercase letters in each column indicate significant differences between treatments (*p* < 0.05). R1–R4 represent garden-cultivated ginseng at 1, 2, 3, and 4 years of cultivation, respectively; R5 represents ginseng cultivated for 20 years in a managed plantation field.

Soil nitrogen dynamics also varied. Ammonium nitrogen (AN) reached its lowest value in R3 (20.8 mg/kg) but was highest in R5 (42.71 mg/kg), suggesting that natural forest conditions favor AN accumulation. Nitrate nitrogen (NN) was highest in R1 (105.33 mg/kg), while other groups showed no significant differences, though R5 had relatively higher NN content. Available potassium (AK) and available phosphorus (AP) followed a fluctuating trend, with R2 and R4 showing higher values, while R3 had the lowest AK (87.66 mg/kg) and AP (1.06 mg/kg). Total nitrogen (TN) followed a “V” trend, peaking in R1 (1.23 g/kg) and reaching a minimum in R3 (0.60 g/kg). Total potassium (TK) was highest in R1 (3.12 g/kg) but declined over time, while total phosphorus (TP) was lowest in R2 (0.54 g/kg) (Table 1). These results indicate that cultivation duration significantly influenced TN, while TK and TP were moderately affected by cultivation years.

### 3.2 Growth characteristics and total ginsenoside content

Ginseng growth characteristics varied significantly with cultivation duration and methods (Table 2). Single plant weight increased progressively from R1 (0.67 g) to R4 (19.04 g), with R5 (2.35 g) being comparable to R2. Main root weight proportion decreased with cultivation years, while fibrous root weight proportion increased, reaching the highest value in R4 (32.26%). Similarly, total length of ginseng increased with time, with R4 (22.64 cm) being the longest, whereas R5 showed a length pattern comparable to R3 (Table 2). The proportion of the main root decreased over time, while the fibrous root proportion increased, indicating a shift in biomass allocation.

Ginsenoside accumulation varied significantly among different cultivation treatments, indicating that both cultivation duration and environmental conditions influence secondary metabolite biosynthesis in ginseng. The mean ginsenoside content was highest in the natural forest understory (R5) treatment (46.48 mg/g), while the lowest was observed in the shortest cultivation duration (R1, 13.96 mg/g). A significant increase in ginsenoside content was observed between R1 and R4 (*p* = 0.0325) (Table 2), suggesting that extended cultivation promotes secondary metabolite synthesis. However, comparisons between intermediate durations (R2, R3) and the R5 control showed no significant differences (*p* > 0.05), implying that ginsenoside accumulation may plateau after prolonged cultivation.

**Table 2:** Single weight, proportion of each part, and ginsenoside content of ginseng in different groups.

Treatment	Single weight(g)	Reed head weight (%)	Main root weight (%)	Fibrous root weight (%)	Ginsenoside (mg/g)
R1	0.67 ± 0.30c	10.16 ± 3.89Ba	88.31 ± 5.99Aa	1.53 ± 3.26Cc	13.96 ± 3.46a
R2	2.75 ± 1.25c	8.54 ± 4.45Ca	75.89 ± 8.30Ab	15.58 ± 8.03Bb	33.35 ± 8.09b
R3	8.44 ± 2.41b	5.19 ± 1.65Cb	74.60 ± 12.42Ab	21.31 ± 11.69Bb	40.31 ± 11.17b
R4	19.04 ± 7.17a	4.99 ± 1.43Cb	62.75 ± 9.57Ac	32.26 ± 9.51Ba	42.26 ± 16.56b
R5	2.35 ± 1.40c	8.88 ± 4.70Ca	74.67 ± 8.23Ab	16.45 ± 7.57Bb	46.48 ± 7.61b

Different uppercase letters indicate significant differences between different parts in the same group; different lowercase letters indicate significant differences between groups (P < 0.05). R1–R4 represent garden-cultivated ginseng at 1, 2, 3, and 4 years of cultivation, respectively; R5 represents ginseng cultivated for 20 years in a managed plantation field.

### 3.3 AMF community structure and diversity under different cultivation regimes

**Figure 1:**
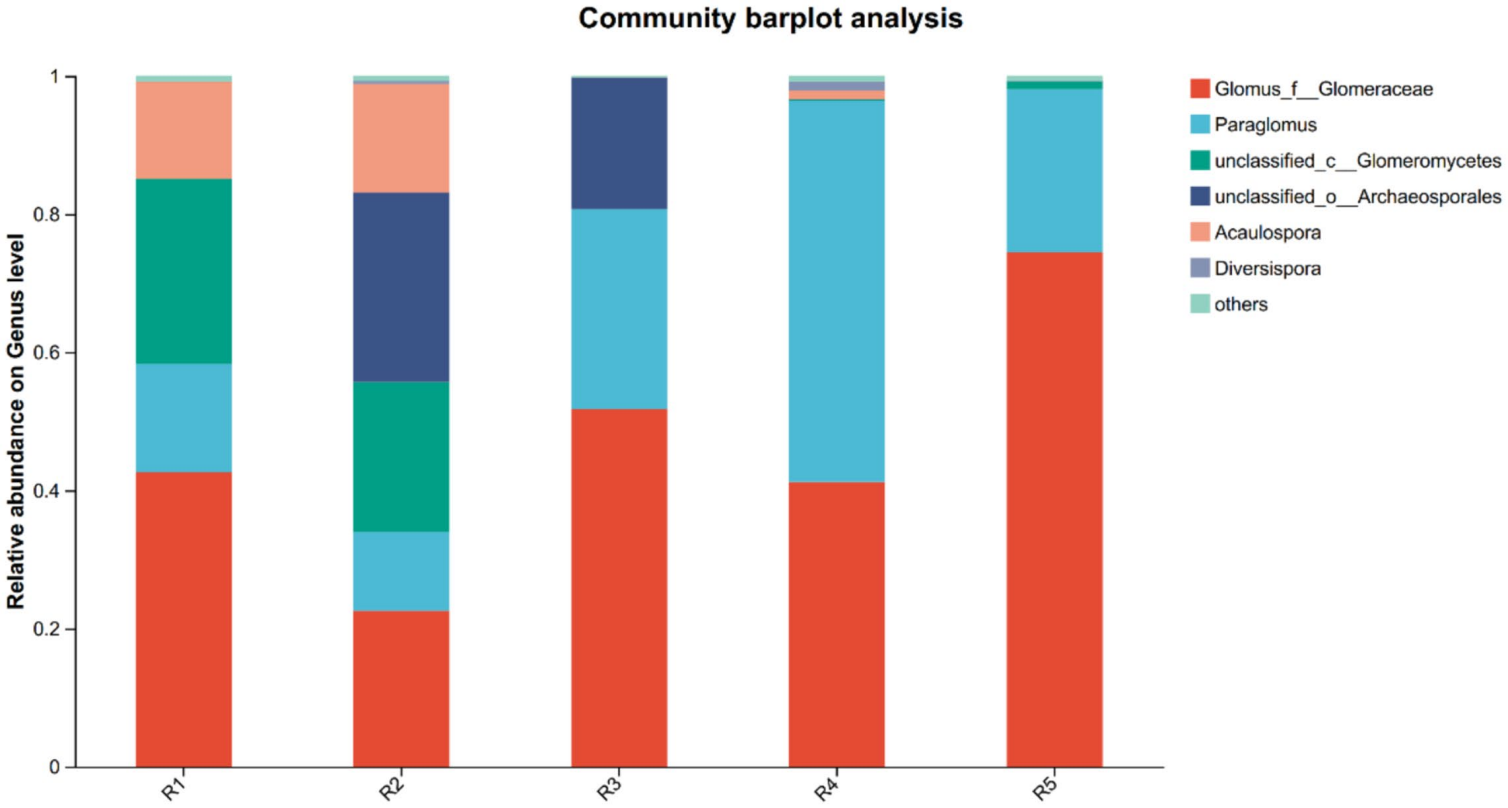
Relative abundance of rhizosphere AMF community of ginseng at the genus level in each group. R1–R4 represent garden-cultivated ginseng at 1, 2, 3, and 4 years of cultivation, respectively; R5 represents ginseng cultivated for 20 years in a managed plantation field.

High-throughput sequencing yielded 567,952 high-quality AMF sequences, with an average read length of 218 bp. Across all samples, *Glomus* and *Paraglomus* were the dominant genera, together accounting for over 95% of relative abundance in both the R4 and R5 groups (Figure 1), indicating their ecological dominance in ginseng rhizospheres regardless of cultivation duration or method.

**Figure 2:**
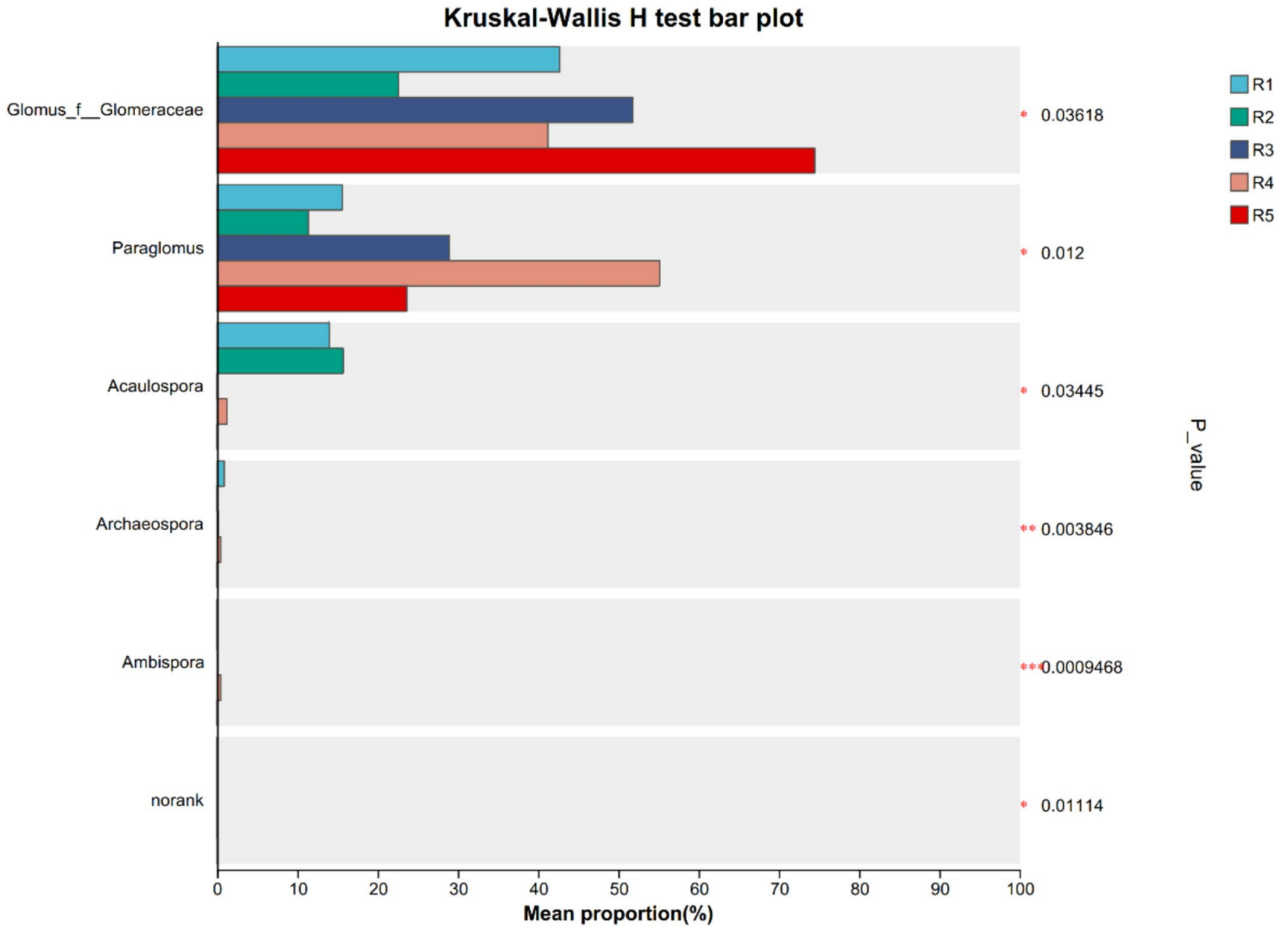
Groups significant differences species among ginseng rhizosphere soil AMF community in each group. ***p < 0.001, **p < 0.01, and *p < 0.05. R1–R4 represent garden-cultivated ginseng at 1, 2, 3, and 4 years of cultivation, respectively, R5 represents ginseng cultivated for 20 years in a managed plantation field.

However, distinct differences in community structure and diversity were observed across cultivation treatments (Figure 2). The number of observed OTUs ranged from 22 (R5) to 35 (R1), with the highest richness detected in early-stage garden cultivation (R1–R2). In contrast, R5 exhibited relatively low OTU richness but high phylogenetic diversity (Pd index), suggesting the presence of evolutionarily distinct AMF taxa (Table 3).

**Table 3:** Index of AMF alpha diversity in the rhizosphere soil of ginseng in each group.

Treatment	Sobs index	Shannon index	Simpson index	Chao 1 index	Ace index	Pielou index	Phylogenetic diversity index
R1	4.40 ± 2.51b	0.54 ± 0.34d	0.69 ± 0.23a	4.40 ± 2.51b	4.40 ± 2.51b	0.39 ± 0.27c	0.90 ± 0.37c
R2	9.00 ± 1.41a	1.58 ± 0.10a	0.25 ± 0.03b	9.00 ± 1.41a	5.80 ± 5.36ab	0.72 ± 0.02a	1.41 ± 0.19ab
R3	8.00 ± 3.54a	1.03 ± 0.58c	0.46 ± 0.29b	8.00 ± 3.54a	7.83 ± 4.56ab	0.49 ± 0.18bc	1.15 ± 0.40bc
R4	10.40 ± 1.34a	1.08 ± 0.19bc	0.43 ± 0.11b	10.60 ± 1.55a	4.86 ± 6.67ab	0.46 ± 0.06bc	1.40 ± 0.07ab
R5	10.40 ± 0.55a	1.49 ± 0.79ab	0.28 ± 0.33b	10.40 ± 0.55a	8.91 ± 5.00a	0.64 ± 0.03ab	1.54 ± 0.11a

Different letters in the same column indicate significant differences between groups for the same index (*p* < 0.05). R1–R4 represent garden-cultivated ginseng at 1, 2, 3, and 4 years of cultivation, respectively; R5 represents ginseng cultivated for 20 years in a managed plantation field.

**Figure 3:**
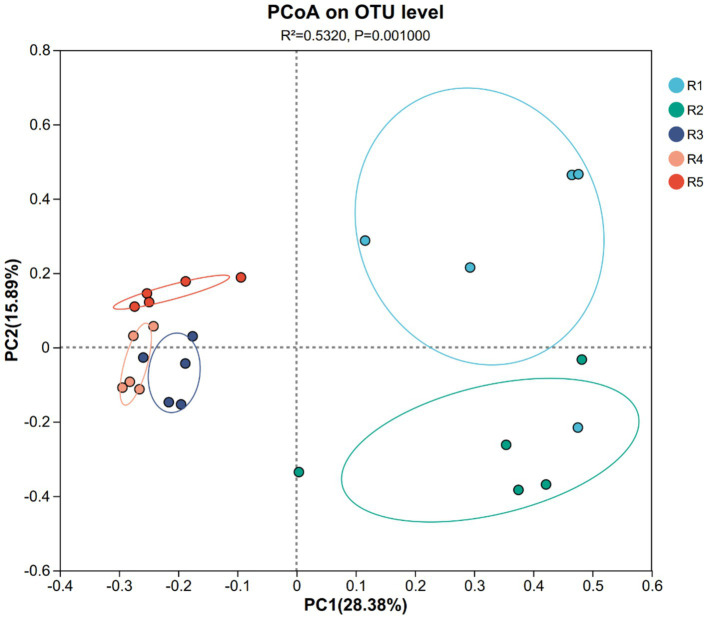
PCoA of ginseng rhizosphere soil AMF community structure in each group. The dots in different colors represent different groups: blue (R1), green (R2), purple (R3), orange (R4), and red (R5). R1–R4 represent garden-cultivated ginseng at 1, 2, 3, and 4 years of cultivation, respectively; R5 represents ginseng cultivated for 20 years in a managed plantation field.

Alpha diversity indices such as Shannon and Pielou’s evenness peaked in R2, reflecting a more complex and balanced AMF community at this intermediate stage. By contrast, R3 and R4 displayed reduced diversity and evenness, likely due to cumulative disturbance from continuous cropping. Principal Coordinates Analysis (PCoA) revealed clear separation between early-stage (R1–R2) and late-stage (R3–R4) garden-cultivated ginseng, with the R5 group forming a distinct cluster (Figure 3), confirming that both cultivation duration and method significantly shape AMF community assembly.

### 3.4 Environmental drivers of AMF community composition and association with ginsenoside accumulation

**Figure 4:**
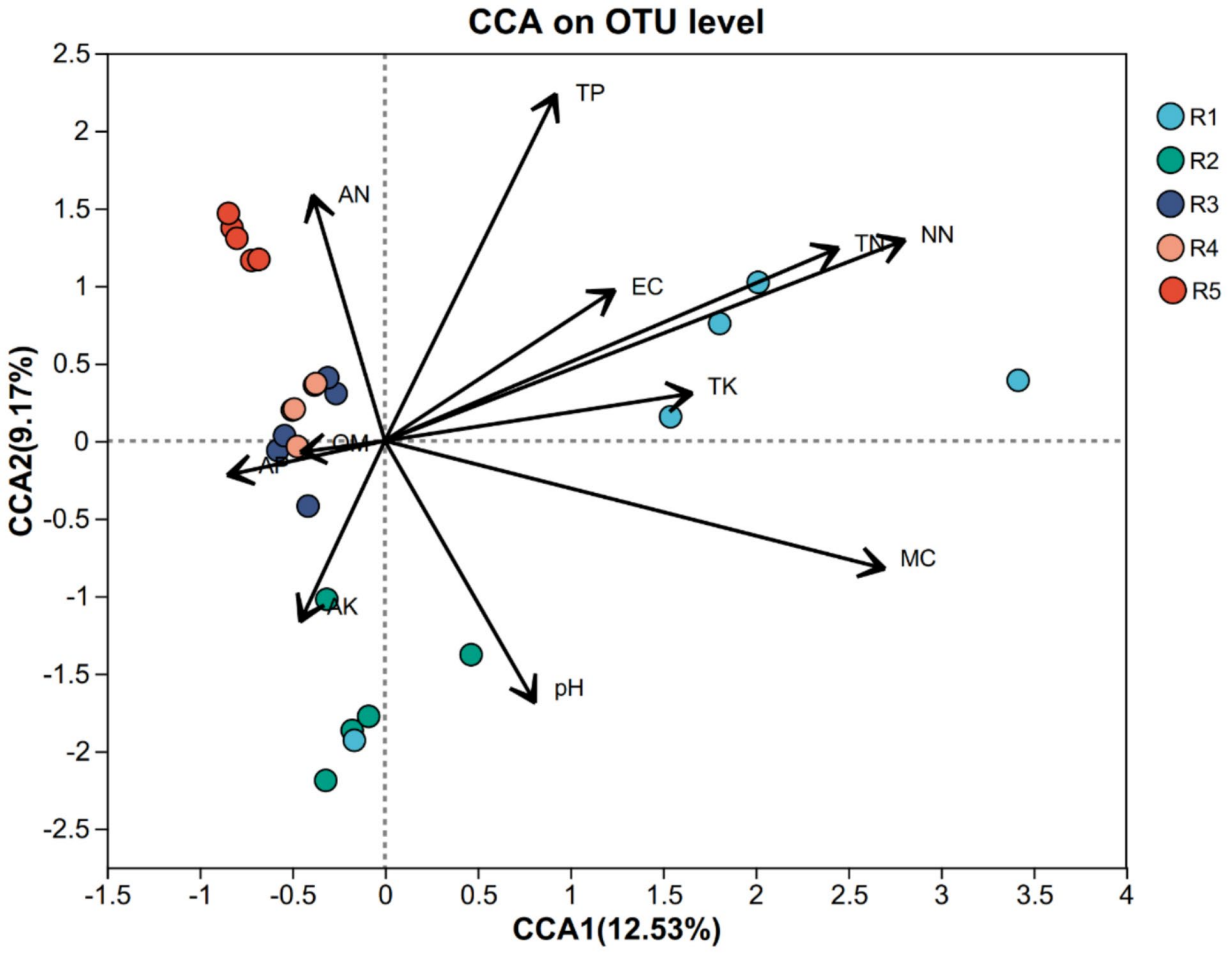
Canonical correspondence analysis (CCA) of AMF community composition at the OTU level constrained by soil physicochemical properties across five cultivation stages (R1–R5). Arrows represent the direction and strength of environmental variables. The length and orientation of arrows indicate their correlation with community variation. Colored dots represent replicate samples from different ginseng cultivation stages: blue (R1), green (R2), purple (R3), orange (R4), and red (R5).

To investigate the influence of environmental factors on AMF community structure, we performed canonical correspondence analysis (CCA) incorporating key soil physicochemical parameters. The ordination revealed that nitrate nitrogen (NN) and total nitrogen (TN) were the most influential variables (r^2^ = 0.7745 and 0.6066, respectively; *p* = 0.001), followed by total phosphorus (TP; r^2^ = 0.4425, *p* = 0.004) and moisture content (MC; r^2^ = 0.6679, p = 0.001) (Figure 4). In contrast, organic matter (OM) and available phosphorus (AP) showed weak correlations with AMF structure (*p* > 0.5), suggesting limited impact in this context.

These findings suggest that nitrogen and phosphorus availability are primary drivers of AMF community assembly, likely due to their role in regulating fungal metabolism and symbiotic efficiency. The negative association between MC and AMF abundance in some groups may indicate moisture stress or competition in drier soils.

Furthermore, correlation analysis revealed a strong positive relationship between AMF diversity indices (e.g., Shannon, Pd) and total ginsenoside content across samples. Specifically, groups with more diverse AMF communities (e.g., R2) tended to exhibit higher levels of ginsenoside accumulation, whereas less diverse or compositionally distinct communities (e.g., R4, R5) were associated with either plateaued or highly variable ginsenoside levels (Tables 2, 3).

These observations suggest that shifts in AMF community structure may indirectly affect secondary metabolism in ginseng, possibly through nutrient modulation or plant signaling pathways. However, we acknowledge that causality cannot be inferred solely from correlations, and future inoculation-based studies are needed to validate these relationships.

## 4 Discussion

The results of this study demonstrate that ginseng cultivation exerts significant effects on soil physicochemical properties and AMF community composition. Continuous cultivation led to notable declines in soil moisture content (MC) and organic matter (OM), with the most pronounced reductions observed in later cultivation stages (R3 and R4). These findings are consistent with previous reports on soil degradation in monoculture systems, where prolonged cultivation depletes essential nutrients and disrupts microbial homeostasis (García et al., 2025; Ortas, 2023). The marked decline in OM in R3 suggests that intensive ginseng farming accelerates nutrient depletion, potentially impairing soil microbial activity and reducing soil fertility over time (Zhan et al., 2024). Given that OM is a critical factor in sustaining soil microbial populations, strategies to enhance organic matter content, such as organic amendments or cover cropping, may be beneficial for long-term ginseng cultivation.

Nitrogen availability emerged as a critical factor influencing AMF community dynamics. Nitrate nitrogen (NN) and total nitrogen (TN) exhibited strong correlations with AMF composition, indicating that nitrogen cycling plays a pivotal role in shaping microbial community structures in ginseng rhizospheres. The high NN levels observed in early cultivation stages (R1) may promote the proliferation of specific AMF taxa, while the subsequent decline in R3 and R4 suggests nutrient exhaustion and potential microbial community shifts (Bossio et al., 2005). Given that AMF are integral to nitrogen acquisition in plants, their community composition may be highly sensitive to fluctuations in soil nitrogen pools (Jiang et al., 2024; Lilleskov et al., 2019). Moreover, total phosphorus (TP) was another key driver of AMF distribution, highlighting the importance of phosphorus availability in sustaining mycorrhizal symbiosis (Ma et al., 2023; Wahab et al., 2023). The significant correlation between TP and AMF diversity suggests that phosphorus fertilization strategies could be optimized to enhance beneficial microbial interactions in ginseng fields.

Soil pH dynamics across different cultivation methods further emphasize the influence of agronomic practices on microbial habitat conditions. The lowest pH recorded in the forest understory (R5) suggests that natural ecosystems may buffer pH fluctuations more effectively than intensive farming systems. Given the moderate correlation between pH and AMF composition (*r*^2^ = 0.2728, *p* = 0.033), future studies should explore the interactive effects of soil acidification, microbial diversity, and nutrient bioavailability on ginseng physiology. Previous studies have suggested that AMF species differ in their pH preferences, with some exhibiting tolerance to acidic soils while others thrive in neutral or alkaline conditions (Kamal et al., 2010; Sinclair et al., 2014; Zheng et al., 2024). Understanding these interactions is crucial for optimizing AMF-mediated soil health management in ginseng cultivation.

The observed shifts in AMF community composition across different cultivation years suggest a progressive transition toward specialized microbial consortia. The clustering patterns in the PCoA analysis indicate that early-stage cultivation (R1–R2) and late-stage cultivation (R3–R4) form distinct microbial assemblages, likely driven by changes in soil nutrient profiles and plant exudation patterns. Such microbial succession phenomena have been documented in various cropping systems, where long-term monoculture fosters the dominance of specific AMF taxa while reducing overall microbial diversity and functional redundancy (Chamberlain et al., 2021; Kurle and Pfleger, 1996; Wang et al., 2025). Notably, the AMF community structure in forest-cultivated ginseng (R5) was markedly different from that in garden-cultivated systems. PCoA results demonstrated that R5 samples formed a distinct cluster, indicating a divergent community assembly process. This divergence may be attributed to the relatively undisturbed soil environment and more heterogeneous microhabitats under forest conditions, which support a more stable and ecologically balanced AMF community (Wang et al., 2015; Zhao et al., 2018). Although *Glomus* and *Paraglomus* remained dominant across all cultivation modes, their relative abundance exceeded 95% in both R4 and R5, suggesting a degree of ecological convergence under conditions of low disturbance (R5) and long-term monoculture stress (R4). However, the R5 group exhibited lower OTU richness and alpha diversity than early-stage garden-cultivated ginseng (R1–R2), possibly due to the presence of natural vegetation, lower nutrient inputs, and differences in root exudation profiles (Filyaw, 2017; Jansa et al., 2014).

Canonical Correspondence Analysis (CCA) further revealed that AMF community composition was significantly influenced by soil physicochemical properties, especially nitrogen and phosphorus availability. Nitrate nitrogen (NN) and total nitrogen (TN) showed strong correlations with community structure, suggesting that nitrogen forms and concentrations are pivotal in shaping AMF assemblages under ginseng cultivation. This aligns with previous findings that elevated nitrogen can alter AMF colonization patterns and symbiotic efficiency (Pan et al., 2020; Zeng et al., 2021). Similarly, total phosphorus (TP) was also a key driver, likely due to the well-documented role of AMF in facilitating phosphorus acquisition in low-P environments (Smith and Read, 2008). Interestingly, organic matter (OM) and available phosphorus (AP) had weak or non-significant associations with AMF community shifts, highlighting that not all nutrient fractions equally impact fungal symbionts.

Together, these findings underscore the complex interplay between soil management, nutrient status, and microbial ecology in ginseng systems. The distinct AMF assemblages observed in forest versus garden cultivation may reflect not only differences in disturbance regimes but also divergent nutrient dynamics, particularly in terms of nitrogen and phosphorus availability. These nutrient-mediated effects likely influence AMF recruitment, diversity, and dominance, thereby shaping plant-microbe interactions and potentially affecting ginseng growth and secondary metabolite accumulation. Further research is warranted to clarify how shifts in AMF structure translate to functional consequences for ginseng health, productivity, and quality under varying cultivation modes.

The observed increase in ginsenoside content with prolonged cultivation suggests that extended ginseng growth enhances secondary metabolite biosynthesis, potentially through improved nutrient acquisition and microbial interactions. Previous studies have reported that longer cultivation periods allow for more extensive root system development, which facilitates nutrient uptake and enhances ginsenoside synthesis (Le et al., 2019). Additionally, the significant difference between R1 and R4, but not between R2, R3, and R5, implies that ginsenoside accumulation may follow a nonlinear pattern, stabilizing after reaching an optimal cultivation duration. Arbuscular mycorrhizal fungi (AMF) have been shown to influence plant secondary metabolism by enhancing phosphorus uptake, modulating hormone signaling pathways, and triggering stress responses that promote ginsenoside biosynthesis (Thokchom et al., 2023; Zhao et al., 2022). The strong correlation between AMF community composition and ginsenoside content further supports this mechanism, suggesting that AMF may serve as key mediators in ginseng quality improvement (Xu et al., 2023). Future studies should focus on elucidating the specific metabolic pathways influenced by AMF and determining the optimal cultivation conditions that maximize ginsenoside accumulation while maintaining sustainable soil health.

## 5 Conclusion

This study demonstrates that the duration and method of ginseng cultivation significantly influence soil physicochemical properties, arbuscular mycorrhizal fungal (AMF) community composition, and ginsenoside accumulation. Key soil factors—moisture content, nitrate nitrogen, total nitrogen, and total phosphorus—were strongly correlated with AMF community structure. Longer cultivation periods led to distinct shifts in AMF diversity and composition, which were positively associated with ginsenoside content. These results suggest that changes in soil conditions and microbial communities may influence ginseng quality.

To improve ginseng yield and pharmacological efficacy, sustainable soil management practices that maintain microbial diversity and nutrient balance are essential. Future research should investigate the functional roles of AMF in ginsenoside biosynthesis and assess microbial-based strategies to optimize ginseng cultivation.

## Data Availability

The data presented in the study are deposited in the SRA repository, accession number PRJNA1255402.
